# Clonal spread of multidrug-resistant *Salmonella* Kentucky ST198 in poultry market environments in Dhaka city, Bangladesh

**DOI:** 10.1371/journal.pone.0342231

**Published:** 2026-04-03

**Authors:** Md Abu Sayem Khan, Mahbub Arefin Rafi, Md Azad Hossen, Md. Majibur Rahman, Sabita Rezwana Rahman

**Affiliations:** Department of Microbiology, University of Dhaka, Dhaka, Bangladesh; Tianjin University, CHINA

## Abstract

*Salmonella enterica* serovar Kentucky ST198 is a globally distributed, multidrug-resistant clone with growing concern due to its zoonotic potential. Despite its detection in Bangladesh from poultry, migratory birds, and fish, genomic data from poultry remain limited which is its main reservoir. This study aimed to characterize poultry-derived *S.* Kentucky ST198 isolates from live bird markets in Dhaka and compare them with strains from migratory birds and fish to assess clonal diversity, resistance profiles, and transmission characteristics. Five isolates recovered from poultry carcasses and slaughterhouse environments from three markets were confirmed as *S.* Kentucky ST198 by whole genome sequencing. All showed resistance to ciprofloxacin, aminoglycosides, sulfonamides, and tetracyclines, supported by the presence of acquired resistance genes (*aadA7, aac(3)-Id, aac(6′)-Iaa, sul1, tet(A), blaTEM-1B*) and mutations in *gyrA* (S83F) and *parC* (S80I). Core-genome SNP analysis revealed that the five poultry isolates differed by only 0–4 SNPs, indicating recent transmission or a shared source within the poultry distribution network. To explore broader ecological links, these genomes were compared with 15 Bangladeshi ST198 genomes from migratory birds and fish. All genomes showed similar antimicrobial resistance and virulence genes profiles, with variable presence of SPI-4, plasmid replicons and integrons. Combined results from pan-genome analysis, core-genome phylogeny, and principal component analysis (PCA) indicated distinct source-associated clustering: poultry isolates formed a tightly clustered, highly clonal group; migratory bird isolates were more dispersed; and fish isolates formed a separate cluster. The open nature of the pan-genome further suggested ongoing gene acquisition within this population. Globally, Bangladeshi isolates formed a monophyletic clade, indicating a locally maintained lineage circulating across environmental and animal reservoirs. Collectively, these findings represent the local circulation of a clonal MDR *S.* Kentucky ST198 lineage across poultry and non-poultry sources and highlight the need for genomic surveillance in high-risk settings such as live bird markets.

## Introduction

*Salmonella* is one of the most common foodborne pathogens causing human gastroenteritis and is frequently associated with foods of animal origin, including poultry, meat, eggs, and their derived products [1]. Globally, *Salmonella* is responsible for an estimated 93 million cases of gastroenteritis and approximately 155,000 deaths each year, with around 85% of these infections linked to foodborne transmission [2]. Non-typhoidal *Salmonella* (NTS) infections are reported worldwide, although prevalence rates vary by region. The species *Salmonella enterica* comprises more than 2,500 known serovars, most of which are motile and zoonotic. Approximately 10% of these serovars have been isolated from poultry [3]. The treatment of severe *Salmonella* infections often relies on third-generation cephalosporins and fluoroquinolones; however, resistance to these critical antimicrobials is increasingly reported [4]. In response, the World Health Organization has categorized fluoroquinolone- and cephalosporin-resistant *Salmonella* among high-priority antimicrobial-resistant (AMR) pathogens [5].

Periodic shifts in the population dynamics of NTS in poultry can lead to the emergence and re-emergence of serovars capable of causing human infections [6]. Among these, *Salmonella enterica* serovar Kentucky is now recognized as an emerging public health concern and is increasingly linked to human infections [7]. *S.* Kentucky is considered a polyphyletic serovar, meaning it includes multiple sequence types (STs) that are genetically diverse and do not share a recent common ancestor. These different STs are thought to vary in their preferred hosts, ecology, and geographical distribution [8,9]. *S.* Kentucky was first reported from poultry in the United States in 1937 and has since been associated with foodborne infections in many parts of the world, particularly through contaminated poultry. Over the past two decades, ciprofloxacin-resistant *S.* Kentucky strains have been detected in Africa, Europe, North America and South Asia [6]. ST198, in particular, is known for its multidrug resistance (MDR), which is largely due to the presence of *Salmonella* Genomic Island 1 (SGI-1). This mobile element carries genes, conferring resistance to several commonly used antibiotics, including ampicillin, chloramphenicol, streptomycin, sulfonamides, and tetracyclines [10]. In addition, resistance to fluoroquinolones in ST198 is linked to specific mutations in the *gyrA* and *parC* genes, making it even more difficult to treat infections [7].

The clinical significance and zoonotic potential of *Salmonella* Kentucky ST198 have been increasingly recognized in recent years. Although historically associated with poultry, this serovar has now been implicated in a growing number of human infections, often linked to international travel or the global trade of food and animals. A distinct epidemic lineage, *S.* Kentucky ST198-X1, has been reported to disseminate across continents through these routes [11]. In the United States, a surveillance study by Haley *et al*. found that 41% of human *S.* Kentucky isolates were from patients with recent international travel, and over half of the isolates shared genomic features with strains from outside the country [12]. Similarly, in South Korea, ciprofloxacin-resistant *S.* Kentucky infections have been closely associated with travel to Southeast Asia [13]. A case report from Belgium further illustrated the clinical impact of ST198, describing treatment failure due to high-level ciprofloxacin resistance and subsequent acquisition of resistance to extended-spectrum cephalosporins and co-trimoxazole [14]. Beyond travel-associated cases, ST198 poses broader concerns due to its multidrug resistance and environmental persistence. Genomic surveillance in China identified extensively drug-resistant ST198 strains from both human clinical samples and the poultry supply chain, with resistance extending to ciprofloxacin, extended-spectrum cephalosporins, and even tigecycline [15]. In the same setting, more than 60% of isolates from poultry and human sources exhibited fluoroquinolone resistance [16]. Additionally, recent studies from the United States have reported nosocomial transmission of ciprofloxacin- and ceftriaxone-resistant ST198 strains between wildlife and hospital environments, indiacting its ability to persist and spread across ecological boundaries [17]. Collectively, these findings underscore the capacity of *S.* Kentucky ST198 to circulate within food production systems, breach species barriers, and cause difficult-to-treat human infections, posing a growing global health challenge.

In Bangladesh, *Salmonella* remains a significant cause of foodborne illness, particularly due to the widespread consumption of poultry and eggs and limited biosecurity in poultry practices. High rates of *Salmonella* contamination have been reported in poultry products, with prevalence estimates ranging from 14% to over 50% [18]. Several studies in Bangladesh have previously reported the occurrence of *S.* Kentucky in poultry. However, these investigations have primarily relied on phenotypic, serological, or PCR-based methods [19,20]. Despite the known zoonotic risk, routine genomic surveillance and molecular epidemiological studies of nontyphoidal *Salmonella* in the country remain limited. Whole genome sequencing (WGS) has emerged as a powerful tool for epidemiological and genomic surveillance. Recent WGS-based studies in Bangladesh have characterized *Salmonella* Kentucky ST198 in non-poultry sources such as migratory birds and fish. However, despite poultry being recognized as a primary reservoir of *S.* Kentucky globally, no WGS-based studies have yet focused on poultry-associated ST198 strains in Bangladesh. This presents a notable gap in understanding the genomic features and public health risks of this high-risk clone in a key transmission setting. Given the country’s high population density, rapid urbanization, and intensive poultry production systems, the local circulation of MDR *S.* Kentucky ST198 may have potential implications for both animal and human health.

In this study, we conducted a whole genome-based investigation of *S.* Kentucky ST198 isolated from poultry market environments in Dhaka, Bangladesh. We compared our poultry-derived genomes with publicly available ST198 genomes previously reported from migratory birds and fish in Bangladesh to assess local clonal diversity. Additionally, to place the Bangladeshi isolates in a broader context, we included *S.* Kentucky ST198 genomes from various geographic regions and sources including poultry, humans, livestock, and food. Our objectives were to characterize the genomic features of these isolates, examine their relatedness to other local and global ST198 strains, and generate baseline genomic data for poultry-derived ST198 in Bangladesh.

## Materials and methods

### Sample collection

A total of 60 fresh chicken carcasses and slaughterhouse environment samples (floor swabs and processing apparatus) were collected from three poultry markets (Kaptan Bazar, Ananda Bazar, and Karwan Bazar) in Dhaka city, Bangladesh between June and August 2023 (S1 Table). As no live animals or human participants were involved, specific ethical approval or animal use permits were not required. All samples were stored in an ice box and immediately transported to the laboratory for processing and isolation of *Salmonella* spp. This study did not involve human participants or animal experimentation. Therefore, ethical approval was not required.

### Isolation and presumptive identification of *Salmonella* spp

All samples were pre-enriched in 10 mL of Buffered Peptone Water (BPW) and incubated overnight at 37 °C. Subsequently, 1 mL of the pre-enriched suspension was transferred to 9 mL of Selenite-Cysteine Broth for selective enrichment and incubated for 18–20 hours at 37 °C. The enriched samples were then serially diluted (10 ⁻ ¹ to 10 ⁻ ⁶) in normal saline, and 100 µL of each dilution was spread onto Xylose Lysine Deoxycholate (XLD) agar plates. After overnight incubation at 37 °C, the plates were examined for *Salmonella*-like red colonies with black centers on XLD agar. Pure cultures were obtained by streaking a single colony onto fresh XLD and Nutrient agar plates. Each isolate was then subjected to a series of biochemical tests, including IMViC (Indole, Methyl Red, Voges-Proskauer, and Citrate utilization), oxidase, catalase, urease production, and Kligler’s Iron Agar test, for presumptive identification of *Salmonella* spp.

### Molecular confirmation of *Salmonella* spp

Molecular identification of presumptive *Salmonella* isolates was performed by detecting the *invA* gene using Polymerase Chain Reaction (PCR). Genomic DNA was extracted using the boiling method, as described previously [21]. Briefly, freshly grown *Salmonella* cultures in Nutrient Broth were centrifuged at 10,000 rpm for 5 minutes, and the resulting pellet was resuspended in 100 µL of nuclease-free water. The suspension was then boiled at 100 °C for 10 minutes, immediately chilled on ice for another 10 minutes, and centrifuged at 13,000 rpm for 10 minutes. The resulting supernatant was used as the DNA template for PCR.

The PCR reaction mixture (25 µL) consisted of 12.5 µL of 2X Master Mix (Promega, USA), 1 µL each of forward and reverse primers (invA-F: 5′-GTGAAATTATCGCCACGTTCGGGCAA-3′ and invA-R: 5′-TCATCGCACCGTCAAAGGAACC-3′) [22], 2 µL of template DNA, and 8.5 µL of nuclease-free water. The amplification was carried out under the following cycling conditions: initial denaturation at 95 °C for 1 minute; 35 cycles of denaturation at 95 °C for 30 seconds, annealing at 64 °C for 30 seconds, and extension at 72 °C for 30 seconds; followed by a final extension at 72 °C for 4 minutes. PCR products were analyzed by electrophoresis on a 1.5% agarose gel.

### Antibiotic susceptibility testing

The Kirby-Bauer disc diffusion method was followed to determine the antibiotic susceptibility of confirmed *Salmonella* isolates to the following antibiotics: Amoxicillin (30 μg), Chloramphenicol (30 μg), Tetracycline (30 μg), Sulfamethoxazole (25 μg), Streptomycin (25 μg), Gentamicin (10 μg), Amikacin (30 μg), Azithromycin (15 μg), Ciprofloxacin (5 μg), Nalidixic acid (30 μg), Meropenem (10 μg), Ceftriaxone (30 μg), and Cefepime (10 μg) (Oxoid, UK). Briefly, normal saline was used to adjust the turbidity of freshly grown *Salmonella* cultures to match the 0.5 McFarland standard. A bacterial lawn was then evenly spread on Mueller-Hinton agar plates, and antibiotic-impregnated discs were placed on the surface. After incubation at 37 °C for 18–20 hours, the diameters of the zones of inhibition were measured and interpreted as susceptible, intermediate, or resistant according to the Clinical and Laboratory Standards Institute (CLSI) 2022 guidelines. *Escherichia coli* ATCC 25922 and *Salmonella Typhimurium* ATCC 14028 were used as control strains.

### Whole genome sequencing, assembly and annotation

Bacterial DNA was extracted using TIANamp bacterial DNA extraction kit according to manufacturer’s instructions. Whole genome sequencing (WGS) was carried out at the International Centre for Diarrhoeal Disease Research, Bangladesh (ICDDR, B), using paired-end 2 × 151 bp reads on the Illumina MiSeq platform. Raw read quality was assessed with FastQC (Version 0.12.1) [23], followed by adapter trimming and quality filtering using Fastp (Version 0.24.1) [24]. The filtered reads were then assembled using UNICYCLER v0.5.1 [25]. Genome assembly characteristics were evaluated with QUAST (Version 5.3.0) [26] and the completeness of the draft genome was assessed using BUSCO (Version 5.8.0) [27]. We used both PROKKA v1.14.6 [28] and Bakta v1.9.4 [29] for the functional annotation of the assembled genomes. Unless otherwise stated, all analyses were performed using default parameters. All the sequences were submitted to the NCBI and deposited under BioProject accession no. PRJNA1283999.

### Characterization of draft genomes

TYGS [30] was used for the taxonomic identification of the assembled genomes. For *in silico* serovar prediction and subtyping, we employed both SeqSero2 v1.3.2 [31] and SISTR v1.1.3 [32]. Multilocus sequence typing (MLST) of the isolates was performed by uploading the assembled genomes to the PubMLST database [33]. AMRFinderPlus v3.12.8 [34] was used to detect antimicrobial resistance (AMR) genes and associated point mutations. Pairwise SNP distances among the sequenced genomes were calculated using SNPs-dists (Version 0.8.2). Salmonella Pathogenicity Islands (SPIs) profiles of genomes were obtained from SPIFinder v2.0 (threshold for minimum % identity = 95%, minimum % coverage = 80%) [35]. Circular genome maps with annotated features were generated using Proksee [12], with *Salmonella* Kentucky PU131 (NCBI accession no CP026327.1) as the reference genome.

### Comparative genomic analysis of *S.* Kentucky ST198 in Bangladesh

In addition to our five sequenced genomes, we retrieved 15 publicly available genomes of *S.* Kentucky ST198 reported from Bangladesh from the NCBI Genome database to perform a comprehensive comparative genomic analysis (S2 Table). Genomes already available in assembled form were directly downloaded in FASTA format. For genomes based on Oxford Nanopore sequencing, raw reads were first subjected to adapter trimming using Porechop (Version 0.2.4) [36], and subsequently assembled using Flye (Version 2.9.6) [37].

The 20 Bangladeshi *S.* Kentucky ST198 genomes were analyzed using a number of bioinformatics tools to profile AMR, virulence factors, mobile genetic elements, SNP distances, and phylogenetic relationships. AMR genes were identified by screening the genomes against ResFinder (Version 4.7.2) [38] and the Comprehensive Antibiotic Resistance Database (CARD) [39]. Virulence genes were detected using the Virulence Factor Database (VFDB) integrated into ABRicate [40]. Draft genomes were also examined for plasmid replicons, insertion sequences, integrons, and prophage sequences using PlasmidFinder (Version 2.1.6) (minimal identity = 95%, minimal coverage = 80%) [41], ISEScan (Version 1.7.2.3) (Remove incomplete IS elements = Yes) [42], BacAnt (Database = IntegronDB)) [43] and PHASTEST [44], respectively, with default parameters.

We also performed a pan-genome analysis using the 20 Bangladeshi genomes with Roary [45]. Draft genomes were first annotated using PROKKA, and the resulting GFF3 files were uploaded to Roary for analysis, using a minimum sequence identity threshold of 95%.

### Phylogenetic analysis

We constructed two phylogenetic trees to compare local and global genomes of *S.* Kentucky ST198 using the same tools and methodologies. The local phylogenetic analysis included 20 genomes characterized earlier in this study. The global analysis incorporated an additional 66 representative genomes retrieved from the NCBI Genome database, encompassing diverse countries and sample sources, resulting in a total of 86 genomes. S3 Table provides the metadata for all genomes used in the global phylogenomic analysis.

For both analyses, *Salmonella* Kentucky PU131 was used as the reference genome. Core-genome alignment and SNP identification were performed using Snippy (version 4.6.0) [46], which calculated the number of single nucleotide polymorphisms (SNPs) between the reference and each genome. Recombination regions were detected and masked using Gubbins (version 3.2.1) [47]. Subsequently, maximum likelihood phylogenetic trees were constructed based on core-genome SNPs using RAxML (version 8.2.12) [48] with the GTRGAMMA substitution model and 1,000 bootstrap replicates. The resulting Newick formatted trees were visualized and annotated with metadata using the Interactive Tree of Life (iTOL) [49].

## Results

### Isolation, molecular identification and antibiotic resistance

Five presumptive *Salmonella* isolates were recovered from the collected samples based on characteristic growth on XLD agar and biochemical characteristics. Isolates designated as SKBD1, SKBD2 were collected from different poultry carcasses samples in Kaptan Bazar, SKBD5 from Kaptan Bazar’s slaughterhouse, SKBD3 from fresh carcasses samples from Ananda Bazar and SKBD4 from Karwan Bazar’s slaughterhouse environment. Molecular confirmation using PCR amplification of the *invA* gene yielded a 284 bp product, confirming their identity as *Salmonella* spp. All isolates exhibited a similar resistance profile, showing resistance to amoxicillin, gentamicin, sulfamethoxazole, ciprofloxacin, amikacin, nalidixic acid, streptomycin, and tetracycline, while susceptible to azithromycin, imipenem, chloramphenicol, ceftriaxone, and cefepime.

### Genomic characteristics of *S.* Kentucky isolates

Whole genome based taxonomic identification of isolates confirmed them as *Salmonella enterica* serovar Kentucky, belonging to ST198 lineage. Subtyping of assembled genome using SeqSero2 and SISTR reaffirmed serotypes as Kentucky and predicted antigenic profile 8:i:z6 (O antigen prediction: 8, H1 antigen prediction(*fliC*): I, H2 antigen prediction(*fljB*): z6).

The genome lengths were consistent across isolates, ranging from 4,804,575–4,807,094 base pairs, with an average GC content of 52.17%. The annotation revealed the number of coding DNA sequences (CDS) between 4,469 and 4,472 (Table 1).

**Table 1:** Genomic characteristics of *Salmonella Kentucky* ST198 isolated from poultry environments in Dhaka, Bangladesh.

Strain	Sequence type	Genome metrics and annotation					Acquired resistance genes	Mutations	
		No. of Contigs	Genome length (bp)	GC content (%)	No. of CDS, rRNA, tRNA, tmRNA	Completeness		*gyrA*	*parC*
SKBD1	ST198	35	4807094	52.17	4470, 3, 79, 1	100%	*aac(3)-Id, aac(6’)-Iaa, aadA7, blaTEM-1B, sul1, tet(A)*	S83F	S80I
SKBD2		38	4806533		4469, 3, 81, 1				
SKBD3		38	4807084		4471, 3, 81, 1				
SKBD4		40	4804575		4471, 2, 71, 1				
SKBD5		38	4806406		4472, 3, 79, 1				

**Fig 1:**
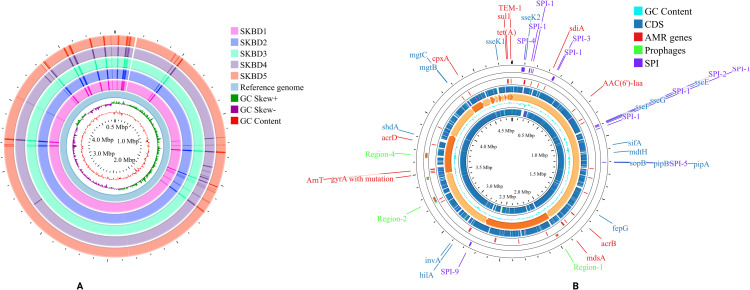
Circular genome visualization of *Salmonella* Kentucky ST198 isolates. **(A)** Whole-genome comparison of five poultry-derived isolates (SKBD1–SKBD5) against a reference genome (PU131). **(B)** Annotated genome of representative isolate SKBD1 showing CDS, AMR genes, SPIs, prophages, and key virulence determinants.

**Fig 2:**
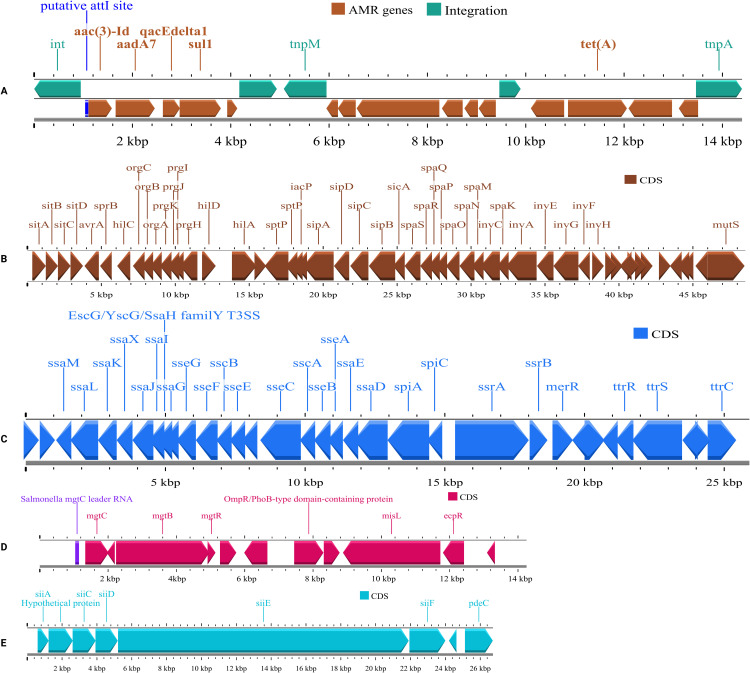
Genetic environments of antimicrobial resistance (AMR) genes and pathogenicity islands (SPIs) in *S.* Kentucky ST198 isolates. **(A)** Genomic region showing co-localization of multiple AMR genes; **(B–E)** Genomic organization of major Salmonella pathogenicity islands: SPI-1 **(B)**, SPI-2 **(C)**, SPI-3 **(D)**, and SPI-4 **(E)**.

All five isolates harbored same acquired antimicrobial resistance genes: *aac(3)-Id*, *aac(6’)-Iaa*, *aadA7*, *blaTEM-1B*, *sul1*, and *tet(A)* (Fig 1). These genes are responsible for resistance to aminoglycosides, β-lactams, sulfonamides, and tetracyclines, aligning with the phenotypic resistance patterns observed. All isolates carried point mutations in the quinolone resistance-determining regions (QRDR) of the *gyrA* (S83F) and *parC* (S80I) genes, which are known to confer resistance to fluoroquinolones. Analysis of the genomic environment of AMR genes revealed the co-localization of *aac(3)-Id*, *aadA7*, *qacE*Δ*1*, *sul1*, and *tet(A)* within a 14-kb region,

clustered alongside a putative integrative element and a mercury resistance operon. In addition, all isolates had sequences encoding four major *Salmonella* pathogenicity islands, harboring numerous virulence genes, including SPI-1 (*hilA, prgH, invG, sipC, spaS, invA*), SPI-2 (*ssaR, ssaV, sseB, ssaE, spiA*), SPI-3 (*mgtC, mgtB, misL, ecpR*), and SPI-4 (*siiA, siiC, siiD, siiE, siiF, pdcC*) (Fig 2). Core SNPs between our poultry derived isolates ranged from 0 to 4 SNP’s.

### Comparative analysis of the Bangladeshi genomes of ST198

Across all 20 genomes, a consistent distribution of AMR genes was observed. *aac(3)-Id*, *aac(6’)-Iaa*, *aadA7*, *blaTEM-1B*, *sul1*, and *tet(A)* were detected in each all of the genomes. However, *aph(3’)-Ia*, another aminoglycoside resistance gene, was present in only 10% of the isolates. No source-specific (poultry, fish, and migratory birds) trends were observed in the AMR gene contents.

**Fig 3:**
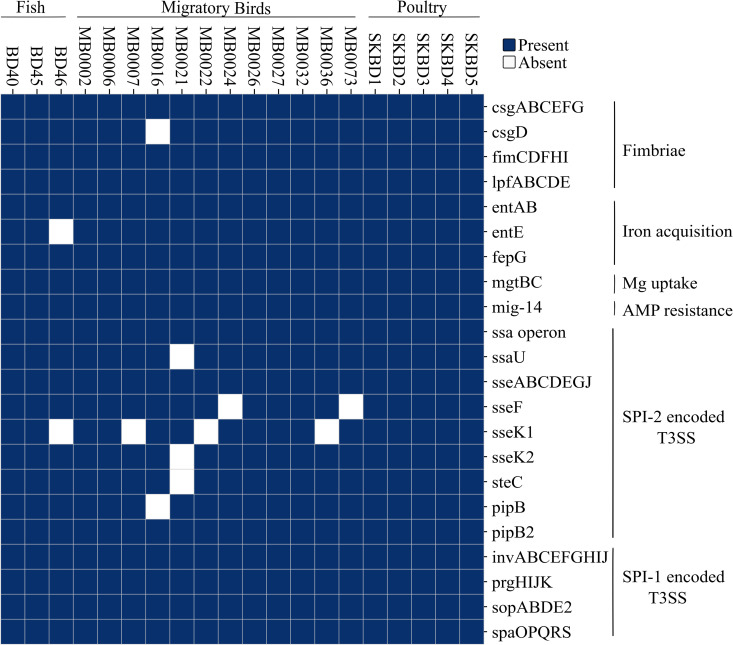
Presence–absence matrix of virulence genes across 20 *S. Kentucky* ST198 genomes from Bangladesh.

All genomes (100%) carried key adhesion and colonization-related genes such as *csgABCEFG*, *fimCDFHI*, *lpfABCDE*, and *mig-14*. Iron acquisition genes (*entAB*, *fepG*, *mgtBC*) and SPI-encoded regulators and effectors, including *prgHIJK*, *invABCEFGHIJ*, *sopABDE2*, *pipB2*, and *ssa*/*sse* operons, were also present in all the genomes (Fig 3). *Salmonella* T3SS effector *sseK1* was present in 75% of the isolates. Only a few genes showed slightly lower frequency due to their absence in specific isolates like, *pipB* in MB0016, *sseK2* and *steC* in MB0021 and *sseF* in MB0024.

**Fig 4:**
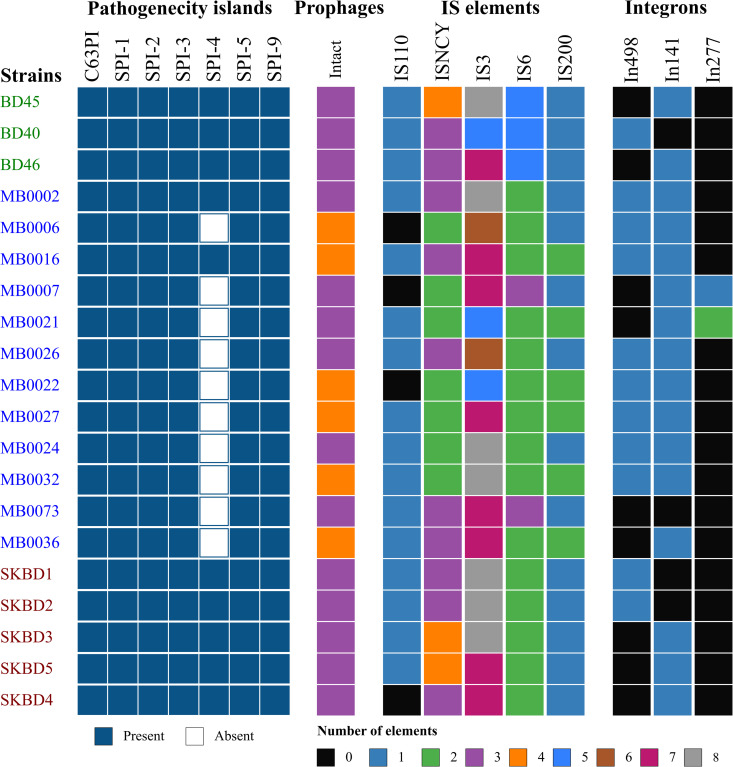
Distribution of mobile genetic elements among *S. Kentucky* ST198 isolates from Bangladesh.

All 20 *Salmonella* Kentucky ST198 isolates harbored the major Salmonella Pathogenicity Islands SPI-1, SPI-2, SPI-3, SPI-5, SPI-9, and C63PI (Fig 4). However, variation was observed in the distribution of SPI-4, which was present in only 50% of the genomes. The number of intact prophages ranged from 3 to 4 per genome (mean: 3.3). The most abundant insertion sequences were IS3 (5–8 copies, mean: 7.0) and ISNCY (2–4 copies, mean: 2.8), followed by IS6 (2–5 copies, mean: 2.6) and IS200 (1–2 copies, mean: 1.3). IS110 was detected in 80% of the isolates, with a presence range of 0–1 copy (mean: 0.8). Among integrons, In141 was found in 80% of the genomes (mean: 0.8 copies), In498 in 55% (mean: 0.6 copies), and In277 in only 10% of isolates.

A total of six plasmid replicon types were identified among the 20 isolates: ColRNAI, IncI1, Col440II, IncQ1, Col156, and Col440I. The most frequently detected replicon was ColRNAI, present in 5 isolates, followed by IncI1 in 4 isolates. Both Col440II and IncQ1 were found in 3 isolates each, while Col156 and Col440I were each detected in a single isolate. Plasmid distribution appeared to be source-associated. All fish-derived isolates carried ColRNAI, Col440II, and IncQ1, while IncI1 was present in 40% of the migratory bird isolates. In contrast, no plasmid replicons were detected in any of the poultry isolates.

**Fig 5:**
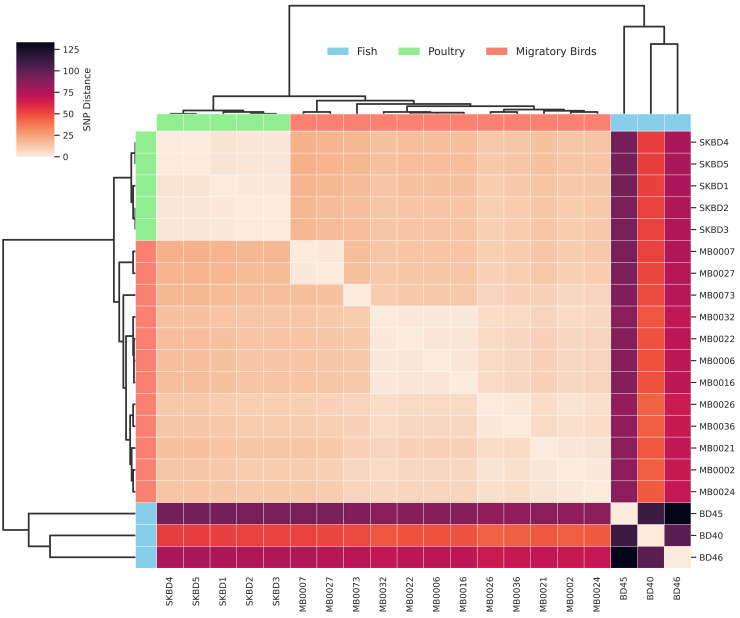
SNP distance matrix of 20 *S. Kentucky* ST198 isolates from Bangladesh.

According to the core genome SNP analysis, the poultry-derived isolates (SKBD1–SKBD5) were highly clonal, differing by only 0–4 SNPs (Fig 5). Migratory bird isolates exhibited low to moderate variation, with pairwise distances ranging from 1 to 16 SNPs. In contrast, the fish-associated isolates (BD40, BD45, BD46) showed greater diversity, differing by 101–133 SNPs. Between-source comparisons showed that poultry isolates were more closely related to migratory bird isolates, with SNP distances ranging from 10 to 20 SNPs. In comparison, poultry and fish isolates differed by 52–94 SNPs, while migratory bird and fish isolates differed by 44–92 SNPs.

**Fig 6:**
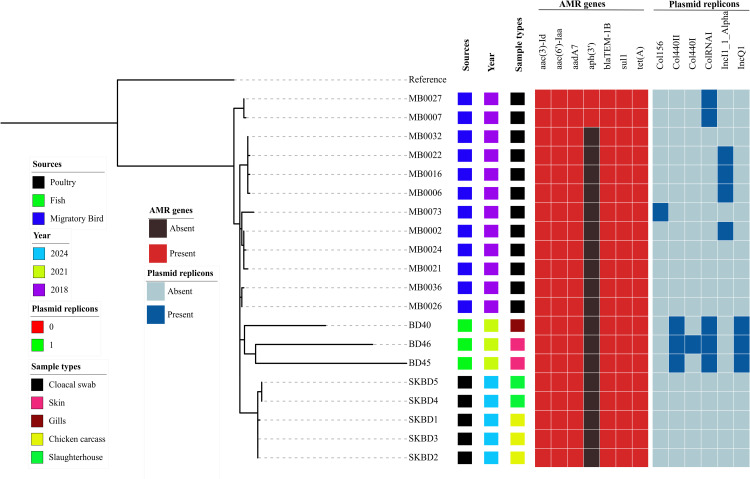
Core genome SNP-based phylogeny of *S. Kentucky* ST198 isolates from Bangladesh.

Core genome SNP-based phylogenetic analysis, midpoint rooted with a reference genome, indicated clustering patterns that corresponded to their ecological sources. All five poultry isolates grouped tightly together with short branch lengths, indicating high clonality (Fig 6). This poultry cluster was closely positioned near a subset of migratory bird isolates, including MB0002, MB0021, MB0024, and MB0073, suggesting genetic relatedness. Other migratory bird isolates such as MB0006, MB0016, MB0022, and MB0032, were distributed more broadly across the tree. The migratory bird isolates (n = 12) exhibited greater genetic variability overall and were distributed across multiple sub-clusters. Fish-derived isolates formed a separate branch on the tree: BD45 and BD46 clustered together, whereas BD40 branched earlier within the same cluster, suggesting within-group diversity among fish isolates. Compared to the poultry and bird isolates, the fish isolates were more distant on the tree, consistent with previously observed SNP distance ranges.

**Fig 7:**
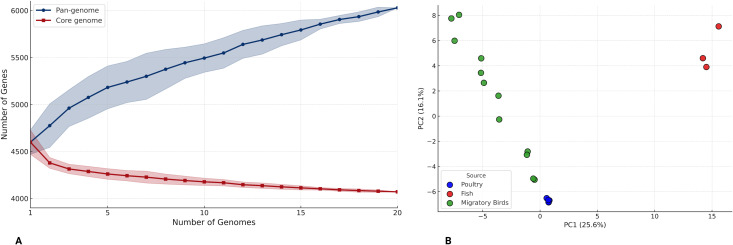
Pan-genome analysis of 20 *Salmonella* Kentucky ST198 genomes from Bangladesh. **(A)** Core and pan-genome rarefaction curve **(B)** Principal component analysis (PCA) based on gene presence–absence matrix.

A pan-genome analysis of 20 *S.* Kentucky ST198 genomes identified 6,029 gene clusters across all genomes. These were categorized as follows: 4,071 (67.5%) core genes shared by ≥99% of isolates, 169 (2.8%) soft-core genes present in 95–98%, 629 (10.4%) shell genes shared by 15–94%, and 1,160 (19.2%) cloud genes found in <15% of isolates. Pan-core rarefaction curve demonstrated a continued increase in the number of total genes as more genomes were added, indicating that the *S.* Kentucky ST198 pan-genome remains open (Fig 7A). The total number of genes expanded from approximately 4,500 to over 6,000 with successive genome additions, while the number of core genes gradually declined and stabilized around 4,100. Further, a Principal component analysis (PCA) was conducted based on gene presence–absence to illustrate diversity among the isolates (Fig 7B). Poultry isolates clustered tightly together in PCA, indicating similar accessory gene profiles. Migratory bird isolates were more widely distributed, with several forming smaller sub-clusters, reflecting greater variability in gene content. Fish-associated isolates formed a distinct group, separated from both poultry and bird isolates, indicating the presence of unique gene sets.

### Phylogenetic relationship of Bangladeshi ST198 in global context

A maximum likelihood phylogenetic tree based on core genome recombination-free SNPs was constructed using 86 *S.* Kentucky ST198 genomes obtained from diverse geographical regions and sources. Isolates from Africa and Asia formed the majority of the diversity, with subclades often segregated by countries or source types. The tree demonstrated zoonotic connections, as human isolates clustered with poultry or food-derived isolates. The close clustering of poultry and environmental isolates suggested localized transmission within Africa. Moreover, some clades showed intercontinental overlap, indicating the global transmission of ST198, likely due to travel or trade. Three North American isolates appeared in separate clades from main cluster, indicating independent introduction events.

**Fig 8:**
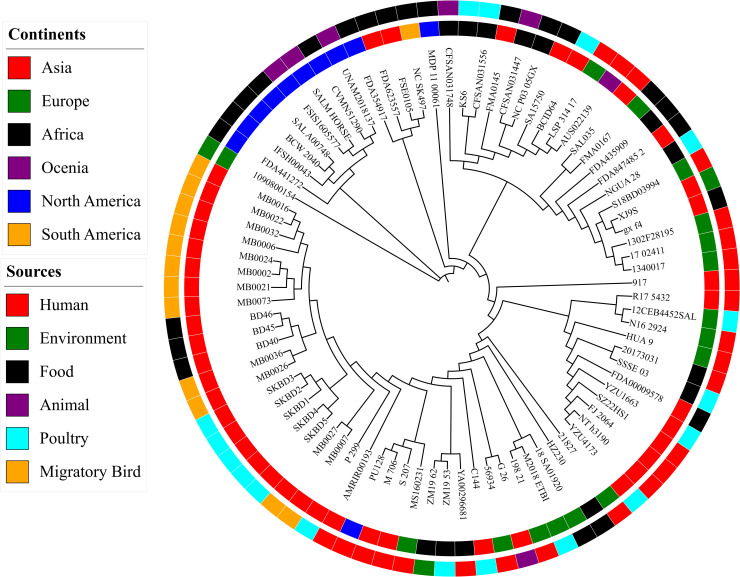
Core genome SNP-based maximum likelihood phylogeny of *S. Kentucky* ST198 isolates from diverse global sources. (The inner ring indicates the continent of origin, while the outer ring shows the source of each isolate.).

In the global phylogenetic context, the Bangladeshi *S.* Kentucky ST198 isolates clustered closely together, forming a monophyletic group that included our poultry market isolates and previously reported strains from migratory birds and fish in Bangladesh (Fig 8). This cluster is separated from other clades representing isolates from other geographical areas including, North America, Europe, and Africa. These findings suggest that the Bangladeshi isolates represent a locally circulating lineage with limited genetic diversity.

## Discussion

Poultry in Bangladesh is a recognized reservoir of *Salmonella*, with multiple studies reporting its presence in live bird markets, particularly in Dhaka [18,50]. These environments facilitate the persistence and dissemination of diverse serovars and thus, pose a potential risk for zoonotic transmission through contaminated meat and handling. Among the commonly detected serovars, *S.* Kentucky has been reported from poultry over different time periods [20,51]. However, the genetic basis of its antimicrobial resistance and transmission characteristics in poultry remains poorly understood due to the limited application of whole genome sequencing (WGS) in poultry-associated surveillance. In this context, we used WGS to characterize *S.* Kentucky ST198 isolates from poultry market environments in Dhaka and compared them with genomes from non-poultry sources (fish and migratory birds) to assess local diversity and transmission patterns.

The poultry-derived *S.* Kentucky ST198 isolates were found to be multidrug-resistant and showed consistent genomic characteristics, carrying the same acquired resistance genes: *aac(3)-Id*, *aac(6’)-Iaa*, *aadA7*, *blaTEM-1B*, *sul1*, and *tet(A)*. These genes corresponded well with the observed phenotypic resistance to aminoglycosides (*aac(3)-Id*, *aac(6’)-Iaa* and *aadA7*), β-lactams (*blaTEM-1B*), sulfonamides (*sul1*), and tetracyclines (*tet(A)*), indicating strong genotype–phenotype concordance. A study from Iran similarly reported 82.3% concordance between phenotypic and WGS-based AMR profiles in poultry *Salmonella* isolates [52]. In addition, the isolates were also resistant to ciprofloxacin due to point mutations in the QRDRs of *gyrA* (S83F) and *parC* (S80I). These specific mutations are commonly found in ciprofloxacin-resistant ST198 isolates from poultry and travel-associated human infections worldwide [13,15]. Notably, the resistance genes *aadA7*, *sul1*, *tet(A)*, and *qacE*Δ*1* were co-occurred within a approximately 14.4kb region alongside an operon responsible for mercury resistance. This structure partially resembles the Salmonella Genomic Island 1 variant (SGI1-K), and a similar genomic environment containing AMR genes has also been observed in East African *S.* Kentucky ST198 isolates [53]. Furthermore, all isolates carried major Salmonella pathogenicity islands (SPIs), including SPI-1, SPI-2, SPI-3, and SPI-4. SPI-1 includes highly conserved genes among *Salmonella* serovars that encode the type III secretion system (T3SS), enabling invasion of the host intestinal epithelium [54]. SPI-2 also encodes a T3SS important for intracellular survival and varies between serovars and even strains [55]. Then, *mgtCB* operon located in SPI-3 encodes the macrophage survival protein MgtC and the magnesium transporter MgtB [56]. The presence of these SPIs suggests the ability of these isolates to invade host cells, survive inside them, and potentially cause disease. This is consistent with virulence patterns reported in international *S.* Kentucky ST198 genomes.

The five *S.* Kentucky ST198 isolates from poultry differed by only 0–4 SNPs, despite being collected from three different live bird markets in Dhaka. This low level of genetic variation suggests a recent common ancestor and local circulation within the poultry market environment. These live bird markets in Dhaka serve as the hub for poultry sourced from all over Bangladesh [57]. A 2012 study on *S.* Kentucky in Bangladeshi poultry farms proposed that shared, non-disinfected transport vehicles and the movement of birds, eggs, and feed between farms and markets could contribute to transmission [20]. Our finding of highly clonal isolates across different markets is consistent with that observation and supports the possibility that contamination and spread are driven by overlapping infrastructure and poor hygiene practices along the poultry supply chain. However, such clonality is consistent with point-source contamination or ongoing transmission within and between market sites, possibly due to shared slaughtering equipment, handling practices, or sourcing from common suppliers. Similar clonal clustering has been documented in other studies where MDR *S.* Kentucky circulateed within integrated poultry production. For example, a study in Jiangsu, China found 1–20 SNP differences among 55 *S.* Kentucky strains collected from an intensive laying hen farm [58]. In a separate study from China, intraregional clonal transmission events were detected, including: (i) 10 chicken isolates from Zhejiang province differing by 0–6 SNPs; (ii) 2 chicken isolates from the same province differing by 4 SNPs; (iii) 2 isolates from pork and duck in Guangxi province with identical genetic backgrounds (0 SNPs) [59]. In clinical settings, three *S.* Kentucky ST198 isolates from a patient with inflammatory bowel disease were also found to be genetically identical, differing by only 2–6 SNPs [60]. These findings collectively indicate that highly clonal *S.* Kentucky strains can circulate in both poultry and humans, often linked to common sources and recent transmission events.

Comparative analysis of Bangladeshi *S.* Kentucky ST198 strains from poultry, migratory birds, and fish indicated a high degree of conserved genomic characteristics. All isolates carried the same set of acquired AMR genes and virulence gene profiles were also consistent. Genes related to adhesion, iron acquisition, and type III secretion systems present in all genomes. In contrast to the conserved core genome and virulence profiles, greater variation was observed in the distribution of mobile genetic elements such as plasmids, pathogenicity islands and integrons. SPI-4 was found in only 50% of the isolates that aligns with the findings of Saraiva et al., who reported SPI-4 in 49.2% of 248 *S.* Kentucky ST198 genomes [53]. All isolates except one (MB0073) carried at least one integron. Previous studies have shown that class 1 integrons, which often carry resistance genes, vary in frequency, structure, and content among *S.* Kentucky and other *Salmonella* serovars [7,61]. In addition, plasmid replicons were more frequently detected in fish-associated isolates and to a lesser extent in migratory bird isolates, whereas poultry isolates lacked detectable plasmids. This suggests that poultry-derived strains might rely more on chromosomally integrated resistance genes, while strains from fish and birds may acquire resistance through mobile elements.

According to the SNP distance and phylogenetic analysis, poultry isolates were highly clonal and related to some migratory bird isolates, indicating potential shared sources or recent transmission. In contrast, fish-derived isolates showed greater genetic divergence and formed a separate cluster, suggesting different origins or independent acquisition events. Similar patterns have been reported elsewhere; for example, *S.* Kentucky ST198 strains from U.S. dairy farms were separated by 66 core-genome SNPs from those isolated in human clinical cases [9]. Pan-genome analysis further supported these patterns, revealing tight clustering of poultry isolates in PCA based on accessory gene content, while bird isolates showed moderate diversity and fish isolates formed a clearly separated group. The overall distribution of core, soft-core, shell, and cloud genes in Bangladeshi ST198 strains was consistent with previous studies [53]. When placed in a global context, all Bangladeshi *S.* Kentucky ST198 isolates formed a single cluster, suggesting the presence of a locally maintained lineage. This lineage belongs to diverse sources, suggesting long-term local persistence and expansion across ecological boundaries. Our data update earlier reports from 2012, which identified a clonally related *S.* Kentucky genotype in commercial poultry farms in Bangladesh [20]. The current detection of ST198 strains in poultry from multiple live bird markets confirmed the continued circulation of that lineage more than a decade later. Importantly, genetically related isolates were also found in fish and migratory birds that typically occupy separate ecological niches. The recovery of ST198 from both poultry and fish at this shared location (Karwan Bazar) strengthens the evidence for localized circulation and potential environmental transmission within the market setting. While *S.* Kentucky is rarely isolated from fish under natural conditions, its presence in wet market samples likely reflects fecal contamination from infected animals or shared water systems [62]. This overlap across species and environments highlights the role of live bird markets as hubs of transmission of ST198, likely driven by unhygienic slaughtering, poor waste management, and interlinked animal supply chains.

The occurrence of ciprofloxacin-resistant *S.* Kentucky ST198 across multiple sources in Bangladesh raises important concerns regarding its zoonotic potential and public health impact. Although whole-genome confirmed human infections have not yet been documented in Bangladesh, a study by Barua et al. (2014) demonstrated that human isolates of *S.* Kentucky ST198 were indistinguishable or closely related to those from poultry based on Pulse Field Gel Electrophoresis (PFGE) analysis, and MLST [3]. This finding supported the notion of zoonotic transmission from poultry to humans within the country. This concern is particularly relevant given the high frequency of human–animal contact in live bird markets and common practices such as the handling of raw poultry and consumption of undercooked products. Internationally, ST198 has been repeatedly associated with human infections, including travel-related cases in Europe and North America, often identified to poultry as the likely source [6,63,64]. A recent study from the United States further reported the adaptability and public health significance of ciprofloxacin-resistant *S.* Kentucky ST198. In that case, an ST198 strain harboring triple QRDR mutations was isolated from wildlife and subsequently from multiple patients and environmental sites within a veterinary hospital, suggesting nosocomial spread [17]. This example underscores the ability of ST198 to persist in complex environments and infect diverse host species, even in high-resource settings. Similarly, the genomic characteristics and clonal nature of our Bangladeshi poultry isolates, along with their detection in migratory birds and fish, suggest that MDR clone circulating in Bangladeshi markets could pose a substantial zoonotic threat.

This study has some limitations. The number of poultry isolates analyzed was relatively small (n = 5), which may not fully represent the broader genomic diversity of *S.* Kentucky ST198 in the poultry sector. In addition, the absence of human or clinical isolates limits our ability to assess possible zoonotic transmission. Despite these constraints, our findings provide important data on the circulation of clonally related strains in poultry and suggest possible ecological connections with non-poultry sources.

## Conclusion

This study presents genome-based characterization of *S.* Kentucky ST198 from poultry in Bangladesh and shows its close genetic relationship to isolates from migratory birds and fish. The detection of a clonal MDR lineage across sources, along with similar resistance and virulence profiles, indicates ongoing local circulation and potential environmental overlap. The presence of genetically related strains in poultry, and fish within shared live bird market environments raises concern for transmission of ST198 and underscores the zoonotic risk associated with these settings. Therefore, continued genomic surveillance is obvious to monitor transmission and inform AMR control strategies within a One Health approach.

## Supporting information

S1 Table Location, types and number of samples collected for this study. (DOCX)

S2 Table List of Bangladeshi *S.* Kentucky ST198 strains used in comparative genomic analysis. (DOCX)

S3 Table Metadata of additional *S.* Kentucky ST198 genomes used in global phylogenetic analysis. (DOCX)
