# Supplementary material for: Clonal spread of multidrug-resistant Salmonella Kentucky ST198 in poultry market environments in Dhaka city, Bangladesh
Source: PLoS One. 2026 Apr 3;21(4):e0342231. doi: 10.1371/journal.pone.0342231 (PMC13048381; doi:10.1371/journal.pone.0342231)
Supplement: S1 Table — (DOCX) [file pone.0342231.s001.docx]

| **Location** | **Sample type** | **No of Samples** | **Total no of sample per location** |
| --- | --- | --- | --- |
| Kaptan Bazar | Poultry carcasses | 13 | 25 |
|  | Slaughterhouse (Floor swab) | 6 |  |
|  | Slaughterhouse (Processing apparatus) | 6 |  |
| Ananda Bazar | Poultry carcasses | 7 | 15 |
|  | Slaughterhouse (Floor swab) | 4 |  |
|  | Slaughterhouse (Processing apparatus) | 4 |  |
| Karwan Bazar | Poultry carcasses | 11 | 20 |
|  | Slaughterhouse (Floor swab) | 4 |  |
|  | Slaughterhouse (Processing apparatus) | 5 |  |

Supplementary table 1: Location, types and number of samples collected for this study
