# Supplementary material for: Clonal spread of multidrug-resistant Salmonella Kentucky ST198 in poultry market environments in Dhaka city, Bangladesh
Source: PLoS One. 2026 Apr 3;21(4):e0342231. doi: 10.1371/journal.pone.0342231 (PMC13048381; doi:10.1371/journal.pone.0342231)
Supplement: S2 Table — (DOCX) [file pone.0342231.s002.docx]

| **Biosample** | **Strain** | **Source** |
| --- | --- | --- |
| SAMN35037656 | MB0002 | Migratory Bird |
| SAMN35037657 | MB0006 | Migratory Bird |
| SAMN35037658 | MB0007 | Migratory Bird |
| SAMN35037661 | MB0016 | Migratory Bird |
| SAMN35037664 | MB0021 | Migratory Bird |
| SAMN35037665 | MB0022 | Migratory Bird |
| SAMN35037667 | MB0024 | Migratory Bird |
| SAMN35037669 | MB0026 | Migratory Bird |
| SAMN35037670 | MB0027 | Migratory Bird |
| SAMN35037672 | MB0032 | Migratory Bird |
| SAMN35037675 | MB0036 | Migratory Bird |
| SAMN35037683 | MB0073 | Migratory Bird |
| SAMN35176374 | BD40 | Fish |
| SAMN35176371 | BD45 | Fish |
| SAMN35176373 | BD46 | Fish |
| This Study | SKBD1 | Poultry |
| This Study | SKBD2 | Poultry |
| This Study | SKBD3 | Poultry |
| This Study | SKBD4 | Poultry |
| This Study | SKBD5 | Poultry |

Supplementary table 2: List of Bangladeshi *S.* Kentucky ST198 strains used in comparative genomic analysis
