# Supplementary material for: Clonal spread of multidrug-resistant Salmonella Kentucky ST198 in poultry market environments in Dhaka city, Bangladesh
Source: PLoS One. 2026 Apr 3;21(4):e0342231. doi: 10.1371/journal.pone.0342231 (PMC13048381; doi:10.1371/journal.pone.0342231)
Supplement: S3 Table — (DOCX) [file pone.0342231.s003.docx]

| **Strain** | **Source** | **Country** |
| --- | --- | --- |
| 917 | Human | Kuwait |
| 21827 | Human | United Kingdom |
| 56934 | Human | United Kingdom |
| 1340017 | Human | United Kingdom |
| 20173031 | Human | South Korea |
| 1090800154 | Enviornment | Netherlands |
| gx-f4 | Food | China |
| 12CEB4452SAL | Poultry | France |
| 1302F28195 | Human | Denmark |
| 17-02411 | Human | Germany |
| 18-SA01920 | Food | Germany |
| 198-21 | Human | Poland |
| ADRDL-NGUA-28 | Poultry | Nigeria |
| AMRIR00193 | Human | India |
| AUSMDU00022139 | Human | Australia |
| BCID64 | Poultry | Indonesia |
| BCW_2040 | Food | USA |
| C144 | Poultry | India |
| CFSAN031447 | Animal | Kenya |
| CFSAN031556 | Poultry | Ethiopia |
| CFSAN031748 | Animal | Ethiopia |
| CVM N51290 | Food | USA |
| FDA00009578 | Food | Vietnam |
| FDA354917 | Food | Mexico |
| FDA435909 | Food | Egypt |
| FDA441272-001-001 | Food | USA |
| FDA623557 | Food | Singapore |
| FDA847485-2 | Food | Jordan |
| FJ-2064 | Human | China |
| FMA0145 | Food | Syria |
| FMA0167 | Food | France |
| FSE0105 | Food | Pakistan |
| FSIS1605577 | Animal | USA |
| G26 | Animal | India |
| HUA_9 | Human | Spain |
| HZ230 | Poultry | Tanzania |
| IFSH00043 | Food | USA |
| KS6 | Poultry | Nigeria |
| LSP_314_17 | Human | Spain |
| M2018_ETBI_003557_01 | Poultry | Hungary |
| M706 | Human | India |
| MDP-11-00061 | Food | USA |
| MS160231 | Human | Ireland |
| N16-2924 | Human | Switzerland |
| NC_P03-05GX | food | Uganda |
| NC_SK497 | Food | Brazil |
| NT-h3190 | Human | China |
| P-299 | Poultry | India |
| PNCS003537 | Human | Canada |
| PU128 | Human | USA |
| R17.5432 | Human | Taiwan |
| S18BD03994 | Human | Belgium |
| S207 | Human | India |
| SA15750 | Poultry | Saudi arabia |
| SAL035 | Human | Saudi arabia |
| SALM-HORSE | Animal | USA |
| SSSE-03 | Poultry | Thailand |
| SZ22HS1 | Human | China |
| UNAM2018137_Sa_AN27 | Animal | Mexico |
| WAPHL_SAL-A00748 | Food | USA |
| XJ9S | Enviornment | China |
| YA00296681 | Human | South Africa |
| YZU1663 | Poultry | China |
| YZU4173 | Poultry | China |
| ZM19-53 | Poultry | Zimbabwe |
| ZM19-62 | Enviornment | Zimbabwe |

Supplementary Table 3: Metadata of additional *S.* Kentucky ST198 genomes used in global phylogenetic analysis
